# Proportion and trend in the age of cigarette smoking initiation among adolescent smoking experiencers aged 13–15 years in 148 countries/territories

**DOI:** 10.3389/fpubh.2022.1054842

**Published:** 2022-11-28

**Authors:** Shuhui Xing, Min Zhao, Costan G. Magnussen, Bo Xi

**Affiliations:** ^1^Department of Epidemiology, School of Public Health, Qilu Hospital, Cheeloo College of Medicine, Shandong University, Jinan, China; ^2^Department of Nutrition and Food Hygiene, School of Public Health, Cheeloo College of Medicine, Shandong University, Jinan, China; ^3^Baker Heart and Diabetes Institute, Melbourne, VIC, Australia; ^4^Research Centre of Applied and Preventive Cardiovascular Medicine, University of Turku, Turku, Finland; ^5^Centre for Population Health Research, University of Turku and Turku University Hospital, Turku, Finland

**Keywords:** cigarette, smoking initiation, adolescents, trends, risk factors

## Abstract

**Background:**

Limited studies have assessed the recent proportion and trend in the age of cigarette smoking initiation among adolescent smoking experiencers globally. We aimed to assess the recent global proportion, associated factors and the long-term trend of the initiated age of cigarette smoking among adolescent smoking experiencers.

**Methods:**

We used data from the most recent Global Youth Tobacco Survey on 99,728 adolescent smoking experiencers aged 13–15 years from 144 countries/territories (hereafter “countries”) that had conducted at least one survey in 2010–20, to assess the recent proportion of the age of cigarette smoking initiation. Additionally, we used data from 148 countries that had conducted ≥2 surveys between 1999 and 2020, to assess the trend in the average age of cigarette smoking initiation.

**Results:**

Among 99,728 adolescent smoking experiencers aged 13–15 years, the proportions of initiating cigarette smoking when aged ≤ 9 years, 10–11 years, 12–13 years, and 14–15 years were 22.8% (95%CI 21.3–24.4), 18.8% (17.3–20.2), 36.8% (34.5–39.2), and 21.6% (19.9–23.2), respectively. The average age of cigarette smoking initiation decreased by 0.44 years per 5 calendar-years averagely in 17 (11.5%) of 148 countries, was unchanged in 95 (64.2%) countries, and increased by 0.38 years per 5 calendar-years averagely in 36 (24.3%) countries. Higher income category (reference group: low-income countries; lower-middle-income: OR = 0.44, 95%CI = 0.28–0.70; upper-middle-income: OR = 0.56, 95%CI = 0.38–0.83; high-income: OR = 0.35, 95%CI = 0.22–0.53) and Framework Convention on Tobacco Control ratification (OR = 0.51, 95%CI = 0.42–0.63) were inversely associated with early cigarette smoking initiation.

**Conclusions:**

A substantial proportion (~80%) of adolescent smoking experiencers aged 13–15 years initiated cigarette smoking before 13 years, and the average age of cigarette smoking initiation decreased or remained unchanged in nearly three quarters of the countries surveyed. These findings emphasize that national governments around the world need to strengthen intervention strategies and measures aimed at children and adolescents to prevent smoking up-take.

## Introduction

Tobacco use is one of the largest causes of preventable deaths, killing 8.7 million people each year and causing tens of millions more to develop preventable disease (1). Based on data from the Global Youth Tobacco Survey (GYTS) conducted in 2010–18, the prevalence of cigarette smoking was 11.3% in boys and 6.1% in girls aged 13–15 years (2), with a large proportion of adolescents who smoke for the first time at a young age becoming regular smokers (3, 4). Smoking early in life is particularly hazardous to health as the neurotoxic effect of tobacco smoke is most pronounced (5). Moreover, cigarette smoking among adolescents is associated with an increase in respiratory symptoms and a decrease in pulmonary function (6), and a higher prevalence of risk factors for cardiovascular disease (7). The health impacts of starting smoking in adolescence do not seem to be contained to that age period. For example, initiating smoking in adolescence can lead to a number of health problems measured 20 years later in adulthood, such as high blood pressure, shortness of breath, and a racing heart (4). The age of initiation in childhood and adolescence also seems to matter, as starting to smoke before age 15 years approximately doubles the risk of premature death in adulthood, with starting to smoke before age 10 years almost three times the excess risk of starting to smoke at age 15 years or older (8).

The health consequences of early smoking initiation aside, adolescence is a key target for smoking prevention with estimates that more than half of adults that smoke initiated this behavior when aged 12–16 years (9). The age of smoking initiation is also suggested to be declining in some regions, from an average age of 22.0 years in 2006 to 17.5 years in 2015 among the Chinese population (10), and from age 18 years before 1970 to age 16 years among males in the 2000s, and from age 19 years to age 15 years among females in Europe (11). However, the age of smoking initiation increased in the United States from an average age of 14.95 years in 2002 to an average age of 16.52 years in 2018 (12). Differences in these trends are not well understood but various factors have been shown to associate with adolescent smoking, including parental smoking (3, 13, 14), and tobacco advertisement and promotion (15). However, there have been limited attempts to determine, at a global level, the age of cigarette smoking initiation, trends in the average age of cigarette smoking initiation, and factors associated with smoking initiation with most previous studies based on information collected retrospectively from adults.

In this study, we assessed the age of cigarette smoking initiation and its associated factors among adolescents aged 13–15 years in 144 countries/territories (hereafter “countries”), using the most recent data collected in the GYTS from 2010–20. We also determined the trend in the average age of cigarette smoking initiation among adolescents in 148 countries from 1999 to 2020.

## Methods

### Study design and participants

The GYTS is a nationally representative, school-based, and cross-sectional survey of adolescents worldwide. It uses a standard two-stage cluster sampling strategy. The schools were randomly selected in the first stage in each country, and then classes were randomly selected within each selected school. All students in the selected classes were eligible to fill in an anonymous and standardized questionnaire, voluntarily. Questionnaires were translated into local languages and then back-translated. The use of standardized methodology and questionnaires in all included countries allows for direct comparisons at the country level. All GYTSs were approved by Local Ethics Committee. More details of the GYTS are available at https://www.who.int/teams/noncommunicable-diseases/surveillance/systems-tools/global-youth-tobacco-survey.

In our study, we used the most recent data from the GYTS conducted in 2010–20 in 144 countries to assess the age of cigarette smoking initiation among adolescents aged 13–15 years. We also used data from 148 countries that conducted two or more GYTSs between 1999 and 2020 to assess trends in the age of cigarette smoking initiation. Participants with complete data on sex, age, and the initiation age of cigarette smoking were included. The flowchart of inclusion/exclusion of related countries is shown in Supplementary eFigure 1.

### Variables

Adolescents smoking experiencers were defined as adolescents aged 13–15 years who had attempted smoking a cigarette in their lifetime, even if only once. The initiation age of cigarette smoking was assessed from the question: “How old were you when you first tried a cigarette?,” with the answers of “I have never tried smoking a cigarette,” “7 years old or younger,” “8 or 9 years old,” “10 or 11 years old,” “12 or 13 years old,” and “14 or 15 years old”. From these responses we categorized the initiation age of cigarette smoking sequentially as 6.5 years old, 8.5 years old, 10.5 years old, 12.5 years old, and 14.5 years old for trend analysis. Parental smoking status was assessed using the question: “Do your parents smoke tobacco?,” with response options of “Neither,” “Father only,” “Mother only,” and “Both.” Tobacco advertisement exposure was defined as a response of “Yes” to any of the following questions: “During the past 30 days, did you see any people using tobacco on TV, in videos, or in movies?,” “During the past 30 days, did you see any advertisements or promotions for tobacco products at points of sale (such as stores, shops, kiosks, super markets, market, restaurant, shops, convenient stores, etc.)?,” and “Do you have something (for example, t-shirt, pen, backpack) with a tobacco product brand logo on it?.” Anti-tobacco advertisement exposure was defined as a response of “Yes” to any of the following questions: “During the past 30 days, did you see or hear any anti-tobacco media messages on television, radio, internet, billboards, posters, newspapers, magazines, or movies?” and “During the past 30 days, did you see or hear any anti-tobacco messages at sports events, fairs, concerts, or community events, or social gatherings?.” Being taught about dangers of tobacco use was defined as a response of “Yes” to the question: “During the past 12 months, were you taught in any of your classes about the dangers of tobacco use?.”

The year of a country ratified World Health Organization Framework Convention on Tobacco Control (WHO FCTC) was obtained from the WHO website. The World Bank classification for the most recent survey year of the GYTS in our study was used to determine the country's income category.

### Statistical analysis

After considering sampling weights, strata, and primary sampling units, proportion estimates and 95% confidence intervals (CI) for the age of cigarette smoking initiation were calculated using SAS version 9.4. The original sampling weights were used to calculate the weighted proportion of the age of cigarette smoking initiation at the country level, and the rescaled weights were used to calculate the overall, regional, and other subgroup estimates, after considering the sample size of each country. Logistic regression analysis was used to examine the factors associated with the age of cigarette smoking initiation. Linear regression was used to assess the trend in the mean age of cigarette smoking initiation over time. A two-sided *P* < 0.05 was considered statistically significant.

## Results

In our study, 144 countries conducted at least one survey in 2010–20. A total of 432,969 adolescents (boys: 50.6%) aged 13–15 years were included, 99,728 (23.0%) of them had their first experience of smoking a cigarette. Twenty four of these 144 countries were from Africa, 30 from the Americas, 24 from the Eastern Mediterranean, 33 from Europe, nine from South-East Asia, and 24 from the Western Pacific (Supplementary eTable 1).

Based on the most recent surveys in 144 countries that conducted at least one survey in 2010–20, 22.8% (95% CI 21.3–24.4) of adolescent smoking experiencers reported their age of cigarette smoking initiation was ≤ 9 years, 18.8% (17.3–20.2) were 10–11 years, 36.8% (34.5–39.2) were 12–13 years, and 21.6% (19.9–23.2) were 14–15 years. The proportion of the age of cigarette smoking initiation was highest in 12–13 years across almost all subgroups (Table 1). However, the proportions of the age of cigarette smoking initiation varied by country (Supplementary eTable 2).

**Table 1:** Proportions of the age of cigarette smoking initiation among adolescent smoking experiencers in 144 countries, 2010–20.

	**Number of countries**	** ≤ 9 years old**	**10–11 years old**	**12–13 years old**	**14–15 years old**
**Total**	144	22.8 (21.3–24.4)	18.8 (17.3–20.2)	36.8 (34.5–39.2)	21.6 (19.9–23.2)
**Age**					
13 years	144	29.6 (25.8–33.4)	25.6 (22.3–28.9)	44.8 (39.6–50.0)	0.0 (0.0–0.0)
14 years	144	21.5 (19.1–23.9)	18.9 (16.5–21.3)	40.5 (36.7–44.2)	19.2 (16.9–21.5)
15 years	144	20.3 (18.0–22.6)	14.9 (12.8–17.1)	29.3 (26.3–32.2)	35.5 (32.3–38.6)
**Sex**					
Boys	144	23.3 (21.3–25.2)	20.0 (18.0–21.9)	37.0 (34.0–39.9)	19.8 (17.8–21.8)
Girls	144	21.9 (19.8–23.9)	16.1 (14.5–17.8)	36.5 (34.0–39.1)	25.5 (23.2–27.7)
**Parental smoking**					
Neither	144	25.6 (23.4–27.7)	18.8 (17.0–20.5)	33.5 (31.7–35.3)	22.1 (20.3–24.0)
Father only	144	22.3 (19.8–24.8)	18.1 (16.2–20.0)	36.3 (33.7–38.9)	23.3 (20.6–25.9)
Mother only	144	21.9 (15.1–28.8)	20.3 (15.2–25.5)	37.0 (30.2–43.8)	20.7 (15.3–26.0)
Both	144	25.0 (19.8–30.1)	19.4 (15.5–23.3)	33.1 (28.9–37.3)	22.5 (18.3–26.7)
**Tobacco advertisement exposure**					
No	144	24.2 (21.5–26.9)	20.5 (17.0–23.9)	33.8 (30.6–37.0)	21.5 (18.4–24.7)
Yes	144	22.5 (20.8–24.2)	18.4 (17.0–19.8)	37.6 (34.8–40.3)	21.6 (19.8–23.3)
**Anti–tobacco advertisement exposure**					
No	144	22.2 (18.7–25.7)	15.8 (13.6–18.0)	40.6 (34.4–46.7)	21.5 (18.5–24.5)
Yes	144	23.1 (21.2–25.0)	20.1 (18.5–21.7)	35.3 (32.8–37.8)	21.6 (19.8–23.3)
**Taught about the dangers of tobacco use**					
No	144	23.2 (20.4–25.9)	17.9 (15.7–20.1)	38.4 (34.1–42.7)	20.5 (18.0–23.1)
Yes	144	22.0 (20.1–23.8)	19.3 (17.5–21.2)	36.1 (33.5–38.6)	22.6 (20.4–24.9)
**WHO region**					
Africa	24	38.0 (34.2–41.7)	21.5 (18.6–24.4)	25.4 (21.9–28.8)	15.2 (12.9–17.5)
Americas	30	13.4 (11.3–15.6)	14.3 (12.3–16.2)	47.0 (42.2–51.9)	25.3 (22.0–28.6)
Eastern Mediterranean	24	23.9 (21.3–26.6)	25.1 (21.6–28.5)	30.6 (27.8–33.4)	20.4 (15.9–24.9)
Europe	33	20.8 (17.9–23.6)	14.4 (13.0–15.9)	39.8 (37.4–42.2)	25.0 (22.6–27.4)
South–East Asia	9	23.5 (19.1–27.9)	20.0 (16.1–23.9)	38.1 (30.7–45.5)	18.3 (15.0–21.6)
Western Pacific	24	20.7 (16.4–25.1)	12.1 (9.9–14.2)	34.2 (30.6–37.8)	33.0 (29.2–36.7)
**World Bank income category**					
Low income	20	35.2 (29.7–40.7)	19.8 (15.2–24.4)	32.0 (23.0–40.9)	13.0 (9.6–16.4)
Lower–Middle income	41	25.7 (23.4–28.0)	21.3 (18.3–24.2)	30.5 (28.1–32.9)	22.5 (18.8–26.1)
Upper–Middle income	48	16.9 (15.2–18.5)	17.6 (15.9–19.2)	42.4 (39.2–45.6)	23.1 (21.0–25.2)
High income	35	12.2 (10.5–13.9)	14.3 (12.8–15.8)	42.0 (39.6–44.4)	31.4 (28.8–34.1)
**FCTC ratification status**					
No	17	21.5 (19.0–24.0)	24.4 (21.3–27.4)	35.0 (31.0–39.1)	19.1 (15.5–22.7)
Yes	127	23.0 (21.3–24.7)	18.1 (16.5–19.7)	37.1 (34.5–39.6)	21.9 (20.1–23.7)

Data are presented as % (95%CI).

WHO, World Health Organization; FCTC, Framework Convention on Tobacco Control.

Higher income category (reference group: low-income countries; lower-middle-income: OR = 0.44, 95%CI = 0.28–0.70; upper-middle-income: OR = 0.56, 95%CI = 0.38–0.83; high-income: OR = 0.35, 95%CI = 0.22–0.53), and countries that had ratified the FCTC (OR = 0.51, 95%CI = 0.42–0.63) were inversely associated with an earlier initiation age of cigarette smoking among adolescent smoking experiencers. Compared to the African region, age of cigarette smoking initiation was later in the regions of South-East Asia (OR = 0.64, 95% CI = 0.44–0.93) and Western Pacific (OR = 0.53, 95% CI = 0.35–0.82) (Table 2).

**Table 2:** Factors associated with age of cigarette smoking initiation among adolescent smoking experiencers in the GYTSs 2010–20.

**Variable**	≤ **13 years old (early) vs. 14–15 years old (late)**^a^	
	***OR* (95%*CI*)**	**P**
**Parental smoking**		
Neither	Ref.	
Father	1.01 (0.85–1.19)	0.928
Mother	1.17 (0.86–1.61)	0.322
Both	1.05 (0.80–1.38)	0.748
**Tobacco advertisement exposure**		
No	Ref.	
Yes	1.02 (0.84–1.24)	0.828
**Anti–tobacco advertisement exposure**		
No	Ref.	
Yes	0.94 (0.82–1.07)	0.329
**Taught about the dangers of tobacco use**		
No	Ref.	
Yes	1.10 (0.97–1.26)	0.134
**WHO region**		
Africa	Ref.	
Americas	0.97 (0.69–1.36)	0.853
Eastern Mediterranean	0.81 (0.56–1.19)	0.280
Europe	0.91 (0.65–1.27)	0.576
South–East Asia	0.64 (0.44–0.93)	0.021
Western Pacific	0.53 (0.35–0.82)	0.004
**World Bank income category**		
Low income	Ref.	
Lower–Middle income	0.44 (0.28–0.70)	0.001
Upper–Middle income	0.56 (0.38–0.83)	0.004
High income	0.35 (0.22–0.53)	< 0.001
**FCTC ratification status**		
No	Ref.	
Yes	0.51 (0.42–0.63)	< 0.001

All variables in the table were introduced into a logistic regression model.

WHO, World Health Organization; FCTC, Framework Convention on Tobacco Control; OR, odds ratio; CI, confidence interval.

a 14–15 years' group is considered as the reference group.

In our study, 148 countries conducted two or more surveys from 1999 to 2020. A total of 363,444 adolescents aged 13–15 years who had first experience of smoking a cigarette were included. During this period, 52 countries conducted two surveys, 45 conducted three surveys, 42 conducted four surveys, seven conducted five surveys, one conducted six surveys, and one conducted eight surveys. Fifteen countries conducted subnational surveys that were used to estimate the average age of cigarette smoking initiation at country level.

**Figure 1:**
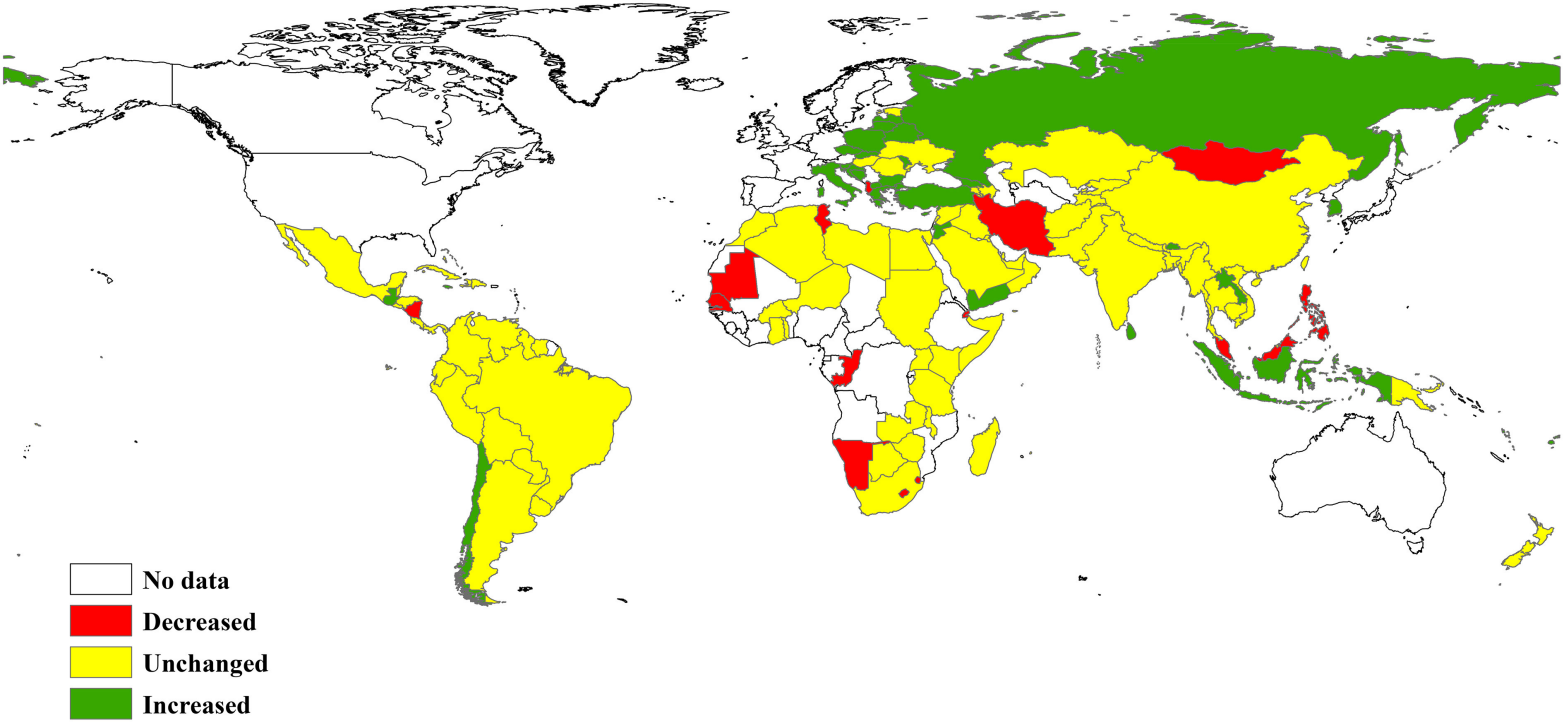
Trend in the age of cigarette smoking initiation among adolescent smoking experiencers between 1999 and 2020.

Overall, the average age of cigarette smoking initiation showed an upward trend over time among adolescent smoking experiencers. Similar upward trends were also observed in most subgroups (Table 3). However, a downward trend was observed in low income and lower-middle income countries, and in the region of Africa. In addition, we found that the average age of cigarette smoking initiation decreased in 17 (11.5%) of 148 countries, was unchanged in 95 (64.2%) countries, and increased in 36 (24.3%) countries (Figure 1 and Supplementary eTable 3).

**Table 3:** Trends in the average age of cigarette smoking initiation among adolescent smoking experiencers between 1999 and 2020.

	**Number of countries**	**First survey, years**	**Last survey, years**	**Absolute change**	**Absolute change per 5–years**	***P* for trend**
**Total**	148	11.07	11.20	0.13	0.06	< 0.001
**Age**						
13 years	148	10.15	10.30	0.15	0.07	0.002
14 years	148	10.90	11.23	0.33	0.15	< 0.001
15 years	148	11.69	11.71	0.02	0.01	< 0.001
**Sex**						
Boys	148	10.99	11.12	0.13	0.06	< 0.001
Girls	148	11.22	11.39	0.17	0.08	< 0.001
**Parental smoking**						
Neither	148	11.01	10.98	−0.03	−0.01	< 0.001
Father only	148	11.13	11.16	0.03	0.01	< 0.001
Mother only	148	11.53	11.40	−0.13	−0.06	< 0.001
Both	148	10.92	10.96	0.04	0.02	< 0.001
**Tobacco advertisement exposure**						
No	148	10.45	11.27	0.82	0.37	< 0.001
Yes	148	11.13	11.19	0.06	0.03	< 0.001
**Anti–tobacco advertisement exposure**						
No	148	11.01	11.38	0.37	0.17	< 0.001
Yes	148	11.15	11.14	−0.01	0.00	< 0.001
**Taught about the dangers of tobacco use**						
No	148	10.98	11.09	0.11	0.05	< 0.001
Yes	148	11.15	11.33	0.18	0.08	< 0.001
**WHO region**						
Africa	23	10.88	10.40	−0.48	−0.22	0.002
Americas	33	11.85	12.09	0.24	0.09	0.003
Eastern Mediterranean	27	10.90	10.91	0.01	0.00	0.125
Europe	32	11.35	11.66	0.31	0.14	< 0.001
South–East Asia	10	10.66	10.88	0.22	0.09	< 0.001
Western Pacific	23	11.60	11.65	0.05	0.03	0.900
**World Bank income category**						
Low income	20	10.63	10.54	−0.09	−0.05	< 0.001
Lower–Middle income	45	10.80	10.78	−0.02	−0.01	< 0.001
Upper–Middle income	50	11.32	11.80	0.48	0.21	< 0.001
High income	33	11.60	12.13	0.53	0.24	< 0.001
**FCTC ratification status**						
No	19	11.25	11.57	0.32	0.17	0.031
Yes	129	11.04	11.14	0.10	0.04	< 0.001

WHO, World Health Organization; FCTC, Framework Convention on Tobacco Control.

## Discussion

Based on the most recent data (2010–20) from 144 countries, nearly 80% of adolescent smoking experiencers aged 13–15 years old initiated cigarette smoking before 13 years old, and the initiated age concentrated on 12–13 years. We found that higher income category and FCTC ratification were inversely associated with early cigarette smoking initiation. In addition, the average age of cigarette smoking initiation decreased or remained unchanged in nearly three quarters of the countries (112/148). Overall, the age of cigarette smoking initiation slightly increased by 0.06 years per 5 calendar-years from 1999 to 2020.

We found that the age of cigarette smoking initiation in low income countries was earlier than lower-middle income, upper-middle income and high income countries. The average age of cigarette smoking initiation decreased in low income and lower-middle income countries. Thus, we need to pay more attention to adolescent smoking in low income areas, especially in African countries, in the future. Our finding also showed that the age of cigarette smoking initiation in countries that had not ratified the FCTC was earlier than those that had ratified the FCTC. The implementation of the FCTC has made substantial gains toward reducing adolescent smoking and smoke exposure in adolescents (16); as have the MPOWER tobacco control measures (17). In addition, we may also pay attention to other tobacco products besides cigarette use in the future, especially the emerging e-cigarette, as E-cigarette use is positively associated with adolescents' initiation of cigarette use (18).

The average age of cigarette smoking initiation decreased or remained unchanged in nearly three quarters the countries observed. Although there was an overall upward trend in the average age of initiation, the rate of increase was slow (increased 0.06 years per 5 calendar-years). In recent decades, the smoking initiation age has decreased dramatically in the Chinese (12), and German populations (19). In our study, a significant proportion of adolescents still started smoking cigarettes before the age of 9 years (22.8%) and from 10–11 years (18.8%) during 2010–20. Of note, the average age of cigarette smoking initiation decreased in low income and lower-middle income countries and region of Africa, which might be due to incomplete implementation of policies against adolescent smoking (20). To prevent adolescents from starting to smoke and to encourage current smokers to quit, more comprehensive and effective tobacco control programs should be developed and fully implemented, especially in the African region.

Our study has several strengths. First, we not only estimated the proportions of the age of cigarette smoking initiation, but we also determined secular trends in the initiation age of cigarette smoking from 1999 to 2020 among adolescents. Second, all countries used the same standardized questionnaire, which allowed comparison by country and region. However, this study has several limitations. First, the GYTS is a school-based survey that included adolescents attending school. As such, our estimates might not be generalizable to adolescents that do not attend school. Second, as the data were self-reported, the results are subject to recall bias. Third, we only included adolescents aged 13–15 years which accounts for the largest proportion of all participants, thus limiting the extrapolation of our results to children or adolescents with other age ranges. Fourth, only the age that the participant first smoked a cigarette was enquired in the questionnaire. Other tobacco products, such as waterpipe, smokeless tobacco, and e-cigarettes were not included. Fifth, 52 countries only conducted two surveys, which might influence our estimates of secular trends. Sixth, as our study used a cross-sectional design, we cannot determine causality in the association between initiation age of cigarette smoking and related factors. Lastly, the trend analysis with linear regression had its limitations in statistical power when data were only from a few points.

## Conclusions

Nearly 80% of adolescent smoking experiencers aged 13–15 years initiated cigarette smoking before the age of 13 years in the period from 2010–20, with the average age that cigarette smoking was initiated decreasing or remaining unchanged in nearly three quarters of the included countries between 1999 and 2020. These findings emphasize the worldwide need to strengthen prevention and intervention efforts aimed at children and adolescents to prevent smoking up-take.

## Data availability statement

The datasets presented in this study can be found in online repositories. The names of the repository/repositories and accession number(s) can be found below: https://www.who.int/teams/noncommunicable-diseases/surveillance/systems-tools/global-youth-tobacco-survey.

## Ethics statement

All GYTSs were approved by Local Ethics Committee. Written informed consent to participate in this study was provided by the participants' legal guardian/next of kin.

## Author contributions

BX designed the study and was the principal investigator. SX performed the data analysis and drafted the first version of the manuscript. BX, MZ, and CM interpreted data and critically revised the manuscript. All authors approved the final version of the manuscript.

## Funding

This study was supported by the Youth Team of Humanistic and Social Science of Shandong University (20820IFYT1902).

## Conflict of interest

The authors declare that the research was conducted in the absence of any commercial or financial relationships that could be construed as a potential conflict of interest.

## Publisher's note

All claims expressed in this article are solely those of the authors and do not necessarily represent those of their affiliated organizations, or those of the publisher, the editors and the reviewers. Any product that may be evaluated in this article, or claim that may be made by its manufacturer, is not guaranteed or endorsed by the publisher.

## References

[1] World Health Organization. Report on the Global Tobacco Epidemic 2021: Addressing New and Emerging Products. San Francisco, CA: University of California at San Francisco, Center for Tobacco Control Research and Education. (2021).

[2] Ma C Xi B Li Z Wu H Zhao M Liang Y. Prevalence and trends in tobacco use among adolescents aged 13-15 years in 143 countries, 1999-2018: findings from the global youth tobacco surveys. Lancet Child Adolesc Health. (2021) 5:245–55. 10.1016/S2352-4642(20)30390-4 33545071

[3] Amiri P Masihay-Akbar H Jalali-Farahani S Karimi M Momenan AA Azizi F. The first cigarette smoking experience and future smoking behaviors among adolescents with different parental risk: a longitudinal analysis in an urban iranian population. Int J Behav Med. (2020) 27:698–706. 10.1007/s12529-020-09910-8 32671634

[4] Amialchuk A Sapci O. The long-term health effects of initiating smoking in adolescence: evidence from a national longitudinal survey. Health Econ. (2022) 31:597–613. 10.1002/hec.4469 34989036

[5] DeBry SC Tiffany ST. Tobacco-Induced Neurotoxicity of Adolescent Cognitive Development (Tinacd): a proposed model for the development of impulsivity in nicotine dependence. Nicotine Tob Res. (2008) 10:11–25. 10.1080/14622200701767811 18188741

[6] Bird Y Staines-Orozco H. Pulmonary Effects of active smoking and secondhand smoke exposure among adolescent students in Juárez, Mexico. Int J Chron Obstruct Pulmon Dis. (2016) 11:1459–67. 10.2147/COPD.S102999 27418819 PMC4934558

[7] Flouris AD Faught BE Klentrou P. Cardiovascular disease risk in adolescent smokers: evidence of a 'smoker lifestyle'. J Child Health Care. (2008) 12:221–31. 10.1177/1367493508092509 18678584

[8] Thomson B Rojas NA Lacey B Burrett JA Varona-Pérez P Martínez MC. Association of childhood smoking and adult mortality: prospective study of 120 000 cuban adults. Lancet Glob Health. (2020) 8:e850–e7. 10.1016/S2214-109X(20)30221-7 32446350 PMC7248573

[9] Nuyts PAW Kuipers MAG Willemsen MC Kunst AE. Trends in age of smoking initiation in the Netherlands: a shift towards older ages? Addiction. (2018) 113:524–32. 10.1111/add.14057 28987013

[10] Pan XB Cao YJ Zhang WH Liu YY. Trends in age of smoking initiation among the Chinese population born between 1950 and 1997. Public Health. (2020) 187:127–33. 10.1016/j.puhe.2020.08.013 32949883

[11] Marcon A Pesce G Calciano L Bellisario V Dharmage SC Garcia-Aymerich J. Trends in smoking initiation in europe over 40 years: a retrospective cohort study. PLoS ONE. (2018) 13:e0201881. 10.1371/journal.pone.0201881 30133533 PMC6104979

[12] Barrington-Trimis JL Braymiller JL Unger JB McConnell R Stokes A Leventhal AM. Trends in the age of cigarette smoking initiation among young adults in the Us from 2002 to 2018. JAMA Netw Open. (2020) 3:e2019022. 10.1001/jamanetworkopen.2020.19022 33021650 PMC7539122

[13] Liao Y Huang Z Huh J Pentz MA Chou CP. Changes in friends' and parental influences on cigarette smoking from early through late adolescence. J Adolesc Health. (2013) 53:132–8. 10.1016/j.jadohealth.2013.01.020 23583505 PMC3691293

[14] Oztekin C Batra M Abdelsalam S Sengezer T Ozkara A Erbas B. Impact of individual, familial and parental factors on adolescent smoking in Turkey. Int J Environ Res Public Health. (2021) 18:740. 10.3390/ijerph18073740 33918478 PMC8038305

[15] Lovato C Watts A Stead LF. Impact of tobacco advertising and promotion on increasing adolescent smoking behaviours. Cochrane Database Syst Rev. (2011) 2011:Cd003439. 10.1002/14651858.CD003439.pub2 21975739 PMC7173757

[16] Prado-Galbarro FJ Auchincloss AH Pérez-Ferrer C Sanchez-Franco S Barrientos-Gutierrez T. Adolescent tobacco exposure in 31 Latin American cities before and after the framework convention for tobacco control. Int J Environ Res Public Health. (2020) 17:7423. 10.3390/ijerph17207423 33053821 PMC7601699

[17] Ünal E Metintaş S. Effectiveness of anti-smoking interventions towards community: a meta-analysis study. Cent Eur J Public Health. (2021) 29:134–42. 10.21101/cejph.a6350 34245554

[18] O'Brien D Long J Quigley J Lee C McCarthy A Kavanagh P. Association between electronic cigarette use and tobacco cigarette smoking initiation in adolescents: a systematic review and meta-analysis. BMC Public Health. (2021) 21:954. 10.1186/s12889-021-10935-1 34078351 PMC8173887

[19] Schneider S Mohnen SM Pust S. The average age of smoking onset in germany–trends and correlates. Int J Public Health. (2008) 53:160–4. 10.1007/s00038-008-6115-4 19127889

[20] Veeranki SP John RM Ibrahim A Pillendla D Thrasher JF Owusu D. Age of smoking initiation among adolescents in Africa. Int J Public Health. (2017) 62:63–72. 10.1007/s00038-016-0888-7 27572496

