# Supplementary material for: Proportion and trend in the age of cigarette smoking initiation among adolescent smoking experiencers aged 13–15 years in 148 countries/territories
Source: Front Public Health. 2022 Nov 28;10:1054842. doi: 10.3389/fpubh.2022.1054842 (PMC9742527; doi:10.3389/fpubh.2022.1054842)
Supplement: Supplementary file 1 [file Data_Sheet_1.doc]

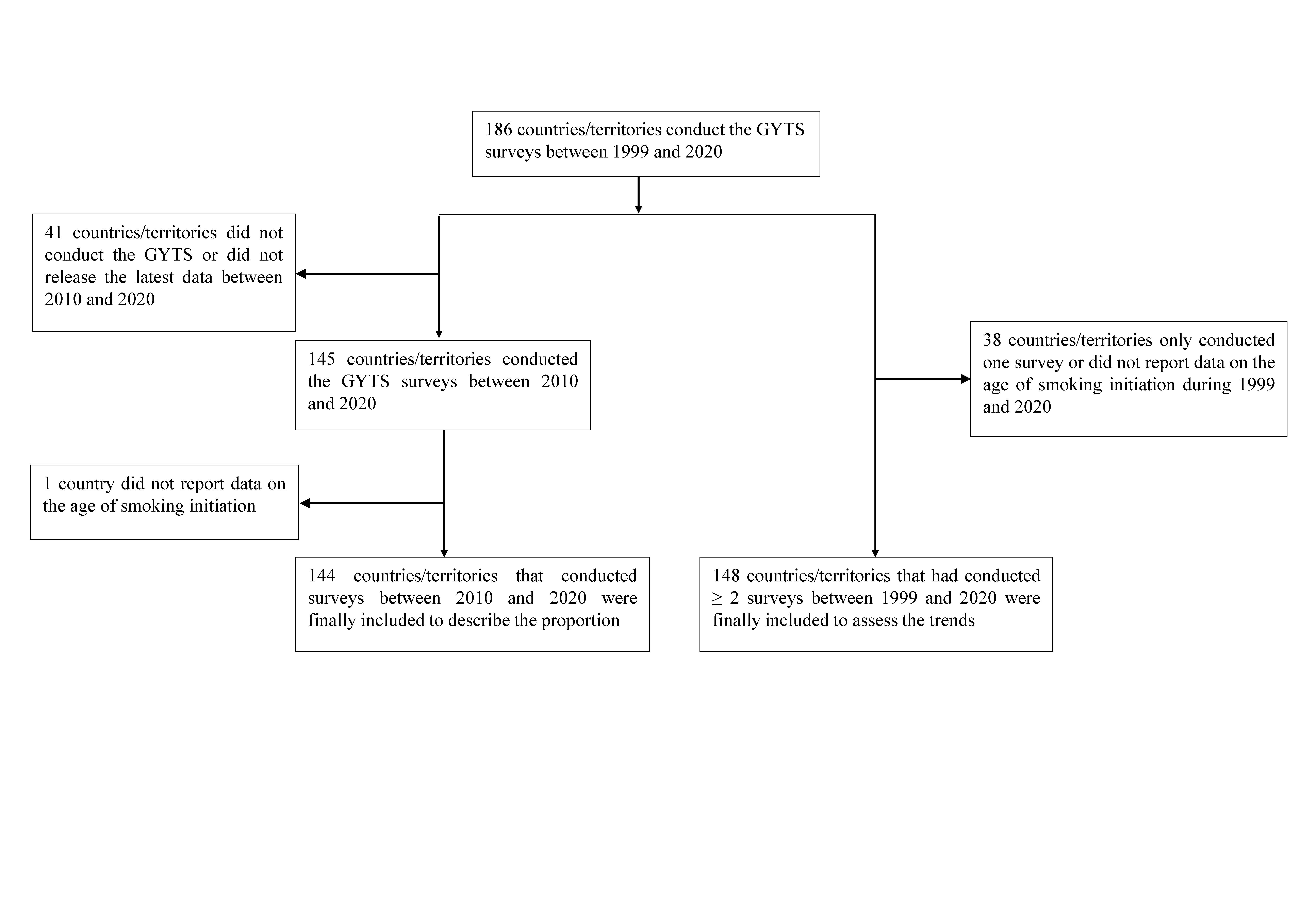


**eFigure 1: Flow chart of inclusion/exclusion of countries/territories. GYTS = Global Youth Tobacco Survey**

**eTable 1.** Characteristics of adolescents aged 13-15 years in 144 countries/territories participating in the Global Youth Tobacco Surveys, 2010-20

| **Country/territory** | **Representativeness** | **Survey year** | **Sample size** | **Smokers** | **Boys, %** | **FCTC ratification Year^*^** |
| --- | --- | --- | --- | --- | --- | --- |
| **Africa** |  |  |  |  |  |  |
| Algeria | National | 2013 | 3952 | 612 | 43.7 | 2006 |
| Angola | Subnational | 2010 | 689 | 16 | 51.1 | 2007 |
| Cameroon | National | 2014 | 1790 | 258 | 53.7 | 2006 |
| Chad | National | 2019 | 870 | 34 | 60.1 | 2006 |
| Comoros | National | 2015 | 1463 | 156 | 45.5 | 2006 |
| Congo | National | 2019 | 3350 | 226 | 49.4 | 2007 |
| Gabon | National | 2014 | 757 | 157 | 46.8 | 2009 |
| Gambia | National | 2017 | 6804 | 873 | 42.9 | 2007 |
| Ghana | National | 2017 | 4738 | 269 | 49.8 | 2004 |
| Kenya | National | 2013 | 1265 | 137 | 48.7 | 2004 |
| Madagascar | National | 2018 | 1490 | 332 | 43.0 | 2004 |
| Mauritania | National | 2018 | 2590 | 363 | 50.3 | 2005 |
| Mauritius | National | 2016 | 3002 | 770 | 48.7 | 2004 |
| Mozambique | National | 2013 | 2898 | 148 | 47.3 | 2017 |
| Sao Tome and Principe | National | 2010 | 3503 | 205 | 44.7 | 2006 |
| Senegal | National | 2020 | 2416 | 158 | 45.3 | 2005 |
| Seychelles | National | 2015 | 1460 | 524 | 48.9 | 2003 |
| Sierra Leone | National | 2017 | 3097 | 219 | 47.8 | 2009 |
| South Africa | National | 2011 | 3739 | 975 | 41.9 | 2005 |
| Togo | National | 2019 | 2172 | 116 | 53.7 | 2005 |
| Uganda | National | 2018 | 1979 | 225 | 48.0 | 2007 |
| United Republic of Tanzania | National | 2016 | 2438 | 81 | 47.8 | 2007 |
| Zambia | National | 2011 | 1674 | 163 | 45.3 | 2008 |
| Zimbabwe | National | 2014 | 4297 | 507 | 48.4 | 2014 |
| **Americas** |  |  |  |  |  |  |
| Antigua and Barbuda | National | 2017 | 1505 | 178 | 49.3 | 2006 |
| Argentina | National | 2018 | 1227 | 431 | 49.9 | Not ratified |
| Bahamas | National | 2013 | 998 | 188 | 53.2 | 2009 |
| Barbados | National | 2013 | 1266 | 284 | 51.0 | 2005 |
| Belize | National | 2014 | 1227 | 277 | 49.4 | 2005 |
| Bolivia | National | 2018 | 3579 | 791 | 49.5 | 2005 |
| Brazil | Subnational | 2012 | 1015 | 226 | 43.8 | 2005 |
| Chile | Subnational | 2016 | 6997 | 2636 | 49.1 | 2005 |
| Costa Rica | National | 2013 | 2113 | 386 | 50.8 | 2008 |
| Cuba | National | 2018 | 3148 | 666 | 50.4 | Not ratified |
| Dominican Republic | National | 2016 | 893 | 99 | 45.9 | Not ratified |
| Ecuador | National | 2016 | 4263 | 1029 | 48.4 | 2006 |
| El Salvador | National | 2015 | 2501 | 693 | 48.7 | 2014 |
| Grenada | National | 2016 | 1571 | 307 | 48.2 | 2007 |
| Guatemala | National | 2015 | 3236 | 1032 | 50.3 | 2005 |
| Guyana | National | 2015 | 942 | 168 | 50.2 | 2005 |
| Honduras | National | 2016 | 2457 | 443 | 47.5 | 2005 |
| Jamaica | National | 2017 | 984 | 281 | 44.3 | 2005 |
| Mexico | National | 2011 | 1757 | 716 | 47.7 | 2004 |
| Nicaragua | National | 2019 | 5320 | 1383 | 48.8 | 2008 |
| Panama | National | 2017 | 2050 | 210 | 50.4 | 2004 |
| Paraguay | National | 2019 | 3385 | 376 | 50.7 | 2006 |
| Peru | National | 2019 | 2527 | 438 | 50.0 | 2004 |
| Saint Kitts and Nevis | National | 2010 | 738 | 120 | 47.6 | 2011 |
| Saint Lucia | National | 2017 | 1199 | 236 | 51.8 | 2005 |
| Saint Vincent and the Grenadines | National | 2018 | 969 | 232 | 47.8 | 2010 |
| Suriname | National | 2016 | 1260 | 346 | 45.7 | 2008 |
| Trinidad and Tobago | National | 2017 | 2213 | 481 | 48.3 | 2004 |
| Uruguay | National | 2014 | 3168 | 758 | 45.9 | 2004 |
| Venezuela | National | 2019 | 5074 | 807 | 48.6 | 2006 |
| **Eastern Mediterranean** |  |  |  |  |  |  |
| Afghanistan | Subnational | 2017 | 1070 | 131 | 56.4 | 2010 |
| Bahrain | National | 2015 | 2354 | 477 | 50.0 | 2007 |
| Djibouti | National | 2013 | 1219 | 137 | 55.4 | 2005 |
| Egypt | National | 2014 | 2047 | 293 | 53.7 | 2005 |
| Gaza Strip | Subnational | 2019 | 1058 | 214 | 46.5 | Not ratified |
| Iraq | National | 2019 | 1585 | 383 | 53.4 | 2008 |
| Jordan | National | 2014 | 1817 | 463 | 50.8 | 2004 |
| Kuwait | National | 2016 | 1997 | 499 | 47.1 | 2006 |
| Lebanon | National | 2011 | 1613 | 403 | 45.7 | 2005 |
| Libyan Arab Jamahiriya | National | 2010 | 1327 | 135 | 48.5 | 2005 |
| Morocco | National | 2016 | 2886 | 217 | 42.3 | Not ratified |
| Oman | National | 2016 | 1466 | 108 | 46.1 | 2005 |
| Pakistan | National | 2013 | 5521 | 388 | 59.2 | 2004 |
| Qatar | National | 2018 | 1562 | 318 | 48.6 | 2004 |
| Saudi Arabia | National | 2010 | 1690 | 380 | 49.3 | 2005 |
| Syrian Arab Republic | National | 2010 | 1149 | 188 | 49.4 | 2004 |
| Tunisia | National | 2017 | 1817 | 389 | 48.8 | 2010 |
| United Arab Emirates | National | 2013 | 3325 | 686 | 50.3 | 2005 |
| UNRWA^a^ GAZA | Regional | 2013 | 1220 | 336 | 53.6 | Not ratified |
| UNRWA^a^ Jordan | Regional | 2014 | 1111 | 318 | 53.1 | Not ratified |
| UNRWA^a^ Lebanon | Regional | 2013 | 1133 | 235 | 45.9 | Not ratified |
| UNRWA^a^ West bank | Regional | 2014 | 1118 | 530 | 43.2 | Not ratified |
| West BANK | Regional | 2016 | 1265 | 504 | 47.4 | Not ratified |
| Yemen | National | 2014 | 1482 | 306 | 60.1 | 2007 |
| **Europe** |  |  |  |  |  |  |
| Albania | National | 2015 | 3407 | 889 | 51.5 | 2006 |
| Azerbaijan | National | 2016 | 2045 | 145 | 53.2 | 2005 |
| Belarus | National | 2015 | 2389 | 706 | 50.6 | 2005 |
| Bosnia and Herzegovina | National | 2013 | 9861 | 3308 | 51.6 | 2009 |
| Bulgaria | National | 2015 | 3446 | 1587 | 51.6 | 2005 |
| Croatia (Hrvatska) | National | 2016 | 2943 | 1312 | 49.4 | 2008 |
| Cyprus | National | 2011 | 733 | 209 | 48.8 | 2005 |
| Czech Republic | National | 2016 | 3482 | 1677 | 51.5 | 2012 |
| Finland | National | 2012 | 4713 | 1875 | 51.1 | 2005 |
| Georgia | National | 2017 | 910 | 208 | 50.9 | 2006 |
| Greece | National | 2013 | 4035 | 1185 | 51.3 | 2006 |
| Italy | National | 2014 | 1505 | 628 | 51.7 | 2008 |
| Kazakhstan | National | 2014 | 1676 | 145 | 49.9 | 2007 |
| Kosovo | National | 2016 | 3730 | 885 | 50.8 | Not ratified |
| Kyrgyzstan | National | 2019 | 5336 | 662 | 50.0 | 2006 |
| Latvia | National | 2019 | 3819 | 1690 | 50.8 | 2005 |
| Lithuania | National | 2018 | 2491 | 1272 | 50.3 | 2004 |
| Macedonia | National | 2016 | 4665 | 1010 | 51.6 | Not ratified |
| Malta | National | 2017 | 1095 | 172 | 55.4 | 2003 |
| Montenegro | National | 2018 | 3787 | 898 | 49.4 | 2006 |
| Poland | National | 2016 | 3776 | 1524 | 50.1 | 2006 |
| Portugal | National | 2013 | 7492 | 2188 | 51.7 | 2005 |
| Republic of Moldova | National | 2019 | 4263 | 1411 | 50.4 | 2009 |
| Romania | National | 2017 | 4287 | 1182 | 51.5 | 2006 |
| Russian Federation | Subnational | 2015 | 5530 | 1851 | 50.4 | 2008 |
| San Marino | National | 2014 | 533 | 101 | 53.3 | 2004 |
| Serbia | National | 2017 | 3292 | 1152 | 49.5 | 2006 |
| Slovakia | National | 2016 | 3502 | 1667 | 50.0 | 2004 |
| Slovenia | National | 2017 | 1928 | 476 | 50.8 | 2005 |
| Tajikistan | National | 2019 | 2925 | 68 | 51.6 | 2013 |
| Turkey | National | 2017 | 82,017 | 21891 | 50.6 | 2004 |
| Turkmenistan | National | 2015 | 6756 | 7 | 49.8 | 2011 |
| Ukraine | National | 2017 | 2962 | 919 | 50.7 | 2006 |
| **South-East Asia** |  |  |  |  |  |  |
| Bangladesh | National | 2013 | 3140 | 140 | 64.5 | 2004 |
| Bhutan | National | 2019 | 2284 | 595 | 49.1 | 2004 |
| Indonesia | National | 2019 | 5030 | 1765 | 48.3 | Not ratified |
| Maldives | National | 2019 | 2867 | 384 | 50.4 | 2004 |
| Myanmar | National | 2016 | 2564 | 520 | 43.9 | 2004 |
| Nepal | National | 2011 | 1537 | 106 | 47.2 | 2006 |
| Sri Lanka | National | 2015 | 1400 | 66 | 49.9 | 2003 |
| Thailand | National | 2015 | 1683 | 439 | 51.0 | 2004 |
| Timor-Leste | National | 2019 | 1529 | 361 | 44.0 | 2004 |
| **Western Pacific** |  |  |  |  |  |  |
| Brunei Darussalam | National | 2019 | 1526 | 251 | 52.2 | 2004 |
| Cambodia | National | 2016 | 1801 | 22 | 48.3 | 2005 |
| Cook Islands | National | 2016 | 306 | 130 | 49.3 | 2004 |
| Fiji | National | 2016 | 1540 | 148 | 48.5 | 2003 |
| Guam | National | 2017 | 1081 | 375 | 51.1 | Not ratified |
| Kiribati | National | 2018 | 1137 | 299 | 47.1 | 2005 |
| Laos | National | 2016 | 3790 | 448 | 48.9 | 2006 |
| Macao (China) | Regional | 2015 | 1174 | 156 | 51.5 | Not ratified |
| Marshall Islands | National | 2016 | 1263 | 229 | 45.4 | 2004 |
| Micronesia | National | 2019 | 3118 | 1004 | 44.6 | 2005 |
| Mongolia | National | 2019 | 3554 | 708 | 50.1 | 2004 |
| New Caledonia | National | 2010 | 571 | 291 | 49.5 | Not ratified |
| Niue | National | 2019 | 66 | 25 | 51.7 | 2005 |
| Northern Mariana Islands | National | 2014 | 1692 | 675 | 51.4 | Not ratified |
| Palau | National | 2017 | 623 | 388 | 50.2 | 2004 |
| Papua New Guinea | National | 2016 | 1227 | 380 | 49.7 | 2006 |
| Philippines | National | 2015 | 5631 | 1388 | 47.5 | 2005 |
| Samoa | National | 2017 | 1000 | 148 | 48.8 | 2005 |
| South Korea | National | 2013 | 3312 | 465 | 51.6 | 2005 |
| Tokelau | National | 2014 | 58 | 45 | 55.1 | Not ratified |
| Tonga | National | 2010 | 1504 | 580 | 39.8 | 2005 |
| Tuvalu | National | 2018 | 415 | 99 | 44.7 | 2005 |
| Vanuatu | National | 2017 | 987 | 151 | 45.8 | 2005 |
| Viet Nam | National | 2014 | 3381 | 267 | 48.8 | 2004 |
| Total | — | — | 432,969 | 99,728 | 50.6 | — |

Data are from <http://www.who.int/fctc/signatories_parties/zh/>.

^a^ United Nations Relief and Works Agency for Palestine Refugees in the Near East.

**eTable 2. Proportions of age of cigarette smoking initiation among adolescent smoking experiencers in 144 countries/territories, 2010-20**

| **Country/territory** | **Smoking initiation** | | | |
| --- | --- | --- | --- | --- |
|  | **≤9 years old** | **10-11 years old** | **12-13 years old** | **14-15 years old** |
| **Africa** |  |  |  |  |
| Algeria | 30.6(26.8-34.4) | 20.1(16.4-23.7) | 29.2(25.3-33.2) | 20.1(15.2-25.1) |
| Angola | 44.2(17.0-71.5) | 8.7(0.0-23.2) | 14.1(0.0-28.5) | 32.9(4.6-61.3) |
| Cameroon | 43.1(36.7-49.4) | 22.7(18.0-27.5) | 18.8(12.6-25.0) | 15.4(10.3-20.5) |
| Chad | 32.4(16.6-48.1) | 23.5(9.3-37.8) | 23.5(9.3-37.8) | 20.6(7.0-34.2) |
| Comoros | 27.2(19.3-35.0) | 20.3(13.8-26.8) | 32.0(25.1-38.9) | 20.5(10.8-30.2) |
| Congo | 43.3(32.2-54.4) | 18.1(11.4-24.9) | 23.7(15.4-32.0) | 14.9(9.2-20.6) |
| Gabon | 22.0(16.3-27.7) | 24.8(16.4-33.2) | 32.8(26.7-38.9) | 20.3(13.6-27.1) |
| Gambia | 33.6(27.6-39.6) | 20.2(16.3-24.1) | 26.1(21.4-30.9) | 20.1(16.4-23.7) |
| Ghana | 41.3(27.7-54.8) | 24.5(14.7-34.4) | 19.8(14.1-25.5) | 14.4(6.6-22.2) |
| Kenya | 42.7(34.9-50.5) | 24.9(17.2-32.5) | 19.7(12.5-26.9) | 12.7(7.5-18.0) |
| Madagascar | 14.1(6.8-21.4) | 19.6(11.0-28.2) | 31.9(23.8-40.0) | 34.5(21.5-47.5) |
| Mauritania | 42.5(35.8-49.3) | 40.6(32.7-48.5) | 10.5(5.3-15.7) | 6.4(2.8-9.9) |
| Mauritius | 14.6(10.8-18.3) | 16.2(11.3-21.0) | 45.6(40.4-50.9) | 23.6(18.7-28.4) |
| Mozambique | 53.0(42.1-63.9) | 16.9(10.8-23.0) | 21.0(14.1-27.9) | 9.1(4.6-13.6) |
| Sao Tome and Principe | 22.9(17.2-28.7) | 23.4(17.6-29.2) | 32.7(26.3-39.1) | 21.0(15.4-26.6) |
| Senegal | 32.3(22.8-41.8) | 22.6(13.0-32.3) | 32.3(23.4-41.2) | 12.8(6.2-19.3) |
| Seychelles | 13.6(9.8-17.4) | 14.0(10.4-17.6) | 42.7(37.8-47.7) | 29.6(23.1-36.2) |
| Sierra Leone | 51.0(37.9-64.2) | 19.3(14.4-24.3) | 17.7(9.4-25.9) | 12.0(5.6-18.3) |
| South Africa | 20.2(15.1-25.3) | 13.7(9.9-17.4) | 31.6(28.0-35.1) | 34.6(28.2-41.0) |
| Togo | 31.1(18.9-43.4) | 22.1(15.0-29.2) | 23.5(13.9-33.2) | 23.2(17.0-29.5) |
| Uganda | 42.6(30.1-55.0) | 23.1(11.9-34.4) | 27.1(17.0-37.3) | 7.2(0.0-15.2) |
| United Republic of Tanzania | 46.8(31.5-62.0) | 20.2(9.4-31.0) | 30.1(16.2-44.1) | 2.9(0.0-5.9) |
| Zambia | 33.7(26.5-41.0) | 15.3(9.8-20.9) | 25.8(19.1-32.5) | 25.2(18.5-31.8) |
| Zimbabwe | 33.6(18.4-48.8) | 15.8(5.6-26.0) | 23.1(16.6-29.7) | 27.5(11.5-43.4) |
| **Americas** |  |  |  |  |
| Antigua and Barbuda | 25.9(19.1-32.7) | 27.6(20.3-35.0) | 30.5(23.9-37.1) | 15.9(10.3-21.5) |
| Argentina | 8.9(4.3-13.5) | 10.8(6.0-15.6) | 56.9(42.4-71.4) | 23.4(16.3-30.5) |
| Bahamas | 29.8(25.5-34.0) | 27.7(20.4-34.9) | 32.6(25.9-39.3) | 10.0(5.8-14.2) |
| Barbados | 23.8(18.5-29.2) | 23.8(18.0-29.6) | 38.2(31.7-44.6) | 14.2(8.9-19.5) |
| Belize | 19.0(13.3-24.8) | 18.8(13.7-23.9) | 33.7(27.9-39.5) | 28.5(23.0-34.1) |
| Bolivia | 20.8(17.4-24.3) | 13.5(11.3-15.8) | 31.4(28.5-34.3) | 34.3(29.3-39.2) |
| Brazil | 18.6(10.9-26.3) | 17.3(11.2-23.3) | 43.3(36.8-49.7) | 20.9(13.7-28.1) |
| Chile | 10.0(8.2-11.8) | 17.4(14.5-20.2) | 46.4(43.9-48.9) | 26.2(23.0-29.5) |
| Costa Rica | 13.3(9.3-17.2) | 17.2(13.2-21.2) | 43.4(37.4-49.4) | 26.1(22.1-30.2) |
| Cuba | 7.6(4.6-10.5) | 16.7(12.7-20.7) | 41.8(37.1-46.6) | 34.0(26.9-41.0) |
| Dominican Republic | 19.3(10.7-27.9) | 19.1(11.5-26.6) | 31.9(19.0-44.8) | 29.7(20.6-38.9) |
| Ecuador | 16.0(13.5-18.5) | 17.8(13.7-21.9) | 39.0(35.6-42.4) | 27.3(21.7-32.8) |
| El Salvador | 16.5(14.0-19.1) | 19.8(16.3-23.3) | 39.2(35.3-43.2) | 24.4(21.0-27.9) |
| Grenada | 28.0(22.8-33.2) | 19.1(13.8-24.4) | 34.4(28.2-40.6) | 18.5(13.1-23.8) |
| Guatemala | 14.2(12.1-16.4) | 14.6(12.2-17.0) | 41.5(37.9-45.0) | 29.6(26.2-33.1) |
| Guyana | 27.9(21.1-34.6) | 18.0(10.3-25.7) | 42.4(33.2-51.6) | 11.7(6.1-17.4) |
| Honduras | 18.6(14.1-23.1) | 16.7(12.1-21.3) | 42.7(37.6-47.9) | 21.9(17.2-26.6) |
| Jamaica | 22.8(17.9-27.7) | 19.6(14.9-24.2) | 32.7(27.3-38.2) | 24.9(19.9-30.0) |
| Mexico | 14.8(11.3-18.3) | 12.7(9.5-15.9) | 55.8(51.4-60.1) | 16.7(13.0-20.5) |
| Nicaragua | 19.9(16.6-23.1) | 17.0(15.1-18.9) | 44.7(41.5-47.9) | 18.4(15.4-21.4) |
| Panama | 15.2(9.4-21.1) | 16.3(10.0-22.6) | 45.4(37.6-53.3) | 23.0(16.7-29.4) |
| Paraguay | 19.3(13.1-25.6) | 12.7(9.7-15.6) | 39.1(34.6-43.5) | 28.9(21.8-36.0) |
| Peru | 10.8(7.2-14.4) | 14.7(10.8-18.7) | 41.4(35.5-47.3) | 33.0(28.2-37.8) |
| Saint Kitts and Nevis | 32.6(24.2-41.0) | 24.6(15.2-34.0) | 30.3(21.5-39.1) | 12.5(4.6-20.5) |
| Saint Lucia | 22.7(17.3-28.1) | 22.3(17.0-27.5) | 38.2(32.1-44.3) | 16.8(10.6-23.1) |
| Saint Vincent and the Grenadines | 25.3(18.5-32.0) | 24.9(18.4-31.3) | 30.8(24.3-37.3) | 19.0(12.8-25.2) |
| Suriname | 23.4(18.2-28.6) | 20.9(16.9-24.9) | 31.7(26.6-36.9) | 24.0(19.2-28.8) |
| Trinidad and Tobago | 21.3(16.7-25.9) | 23.2(18.9-27.5) | 32.2(27.8-36.5) | 23.3(19.0-27.7) |
| Uruguay | 9.5(6.1-12.9) | 15.6(12.0-19.1) | 38.0(31.6-44.4) | 36.9(30.5-43.3) |
| Venezuela | 11.9(6.9-16.9) | 14.2(10.5-18.0) | 44.0(34.0-54.1) | 29.8(17.4-42.2) |
| **Eastern Mediterranean** |  |  |  |  |
| Afghanistan | 37.2(26.8-47.7) | 18.0(10.2-25.8) | 27.1(19.1-35.1) | 17.7(11.3-24.1) |
| Bahrain | 30.9(26.0-35.7) | 18.6(14.7-22.5) | 33.7(29.2-38.2) | 16.8(12.7-21.0) |
| Djibouti | 32.3(22.1-42.5) | 21.2(13.7-28.7) | 29.3(21.3-37.3) | 17.2(7.3-27.1) |
| Egypt | 18.5(10.1-26.8) | 41.5(33.0-50.0) | 32.2(23.6-40.8) | 7.8(1.4-14.2) |
| Gaza Strip | 26.8(22.9-30.7) | 21.3(15.8-26.7) | 33.0(27.2-38.8) | 19.0(13.4-24.5) |
| Iraq | 23.1(17.5-28.7) | 21.6(17.3-26.0) | 33.7(27.9-39.4) | 21.6(18.0-25.2) |
| Jordan | 19.2(15.6-22.8) | 18.6(14.3-23.0) | 39.1(34.6-43.7) | 23.0(17.1-28.8) |
| Kuwait | 20.7(16.7-24.8) | 17.7(13.0-22.5) | 39.3(33.0-45.7) | 22.2(17.3-27.1) |
| Lebanon | 26.7(22.5-30.9) | 21.5(17.8-25.2) | 34.8(30.6-38.9) | 17.1(11.9-22.2) |
| Libyan Arab Jamahiriya | 30.2(21.9-38.5) | 15.8(10.4-21.1) | 32.2(23.8-40.7) | 21.8(13.4-30.1) |
| Morocco | 34.6(27.0-42.2) | 13.0(5.8-20.2) | 24.8(16.1-33.6) | 27.6(17.2-37.9) |
| Oman | 31.9(22.4-41.3) | 15.5(8.6-22.5) | 30.5(21.5-39.5) | 22.1(11.8-32.3) |
| Pakistan | 40.0(27.6-52.3) | 15.7(8.1-23.3) | 32.0(18.7-45.2) | 12.3(6.9-17.8) |
| Qatar | 27.4(21.5-33.2) | 19.5(16.3-22.7) | 33.3(28.6-38.0) | 19.8(14.4-25.2) |
| Saudi Arabia | 21.6(14.8-28.4) | 19.8(15.4-24.1) | 28.0(21.4-34.7) | 30.6(20.6-40.6) |
| Syrian Arab Republic | 21.9(13.7-30.2) | 20.7(12.4-28.9) | 22.9(16.1-29.7) | 34.5(18.8-50.2) |
| Tunisia | 29.9(24.6-35.3) | 15.2(11.3-19.0) | 35.0(29.1-41.0) | 19.9(16.2-23.6) |
| United Arab Emirates | 28.6(25.3-32.0) | 18.3(15.7-21.0) | 33.2(29.5-36.8) | 19.9(16.5-23.3) |
| UNRWA^a^ GAZA | 30.4(25.5-35.2) | 21.7(17.7-25.7) | 31.0(27.1-35.0) | 16.9(10.1-23.7) |
| UNRWA^a^ Jordan | 20.1(15.4-24.8) | 18.4(15.3-21.5) | 39.7(31.6-47.8) | 21.8(15.7-27.9) |
| UNRWA^a^ Lebanon | 20.7(15.0-26.4) | 22.2(15.3-29.2) | 39.8(33.8-45.8) | 17.3(13.0-21.5) |
| UNRWA^a^ West bank | 23.1(18.6-27.6) | 18.2(14.0-22.4) | 41.6(35.8-47.3) | 17.1(12.8-21.5) |
| West BANK | 24.6(20.5-28.6) | 22.4(17.8-27.1) | 30.9(26.1-35.8) | 22.0(15.8-28.3) |
| Yemen | 17.6(12.6-22.6) | 21.9(15.1-28.7) | 32.5(26.3-38.7) | 28.0(21.1-34.9) |
| **Europe** |  |  |  |  |
| Albania | 37.3(32.8-41.8) | 15.8(13.2-18.4) | 25.9(23.0-28.8) | 21.0(17.5-24.5) |
| Azerbaijan | 33.6(25.8-41.3) | 13.6(7.7-19.4) | 32.6(21.8-43.4) | 20.3(11.7-28.8) |
| Belarus | 15.8(13.6-18.1) | 19.6(15.6-23.6) | 37.3(32.9-41.7) | 27.3(21.1-33.4) |
| Bosnia and Herzegovina | 32.3(28.8-35.9) | 17.1(15.5-18.6) | 30.5(28.4-32.6) | 20.1(16.1-24.1) |
| Bulgaria | 15.9(11.9-19.9) | 14.0(10.8-17.2) | 41.8(38.2-45.3) | 28.4(24.3-32.5) |
| Croatia (Hrvatska) | 19.4(16.2-22.6) | 14.7(12.2-17.2) | 37.3(33.8-40.9) | 28.5(23.1-34.0) |
| Cyprus | 13.4(8.8-18.0) | 14.8(10.0-19.7) | 37.3(30.8-43.9) | 34.4(28.0-40.9) |
| Czech Republic | 16.0(13.9-18.0) | 22.2(19.9-24.6) | 47.4(44.7-50.2) | 14.4(12.4-16.3) |
| Finland | 12.4(9.8-15.0) | 20.8(19.3-22.3) | 45.1(42.2-48.0) | 21.7(19.0-24.4) |
| Georgia | 35.5(29.4-41.6) | 13.3(6.0-20.6) | 27.2(21.4-33.0) | 23.9(19.0-28.9) |
| Greece | 18.3(15.5-21.2) | 13.2(11.4-15.0) | 38.5(34.9-42.0) | 30.0(25.5-34.5) |
| Italy | 4.2(2.3-6.1) | 12.0(9.0-15.1) | 50.8(46.5-55.0) | 33.0(27.8-38.2) |
| Kazakhstan | 42.2(29.1-55.2) | 13.1(8.1-18.0) | 27.0(16.5-37.6) | 17.7(9.1-26.4) |
| Kosovo | 29.8(25.5-34.0) | 19.3(15.7-22.9) | 35.4(31.9-38.8) | 15.5(12.6-18.5) |
| Kyrgyzstan | 36.7(28.9-44.5) | 12.2(8.8-15.6) | 26.5(21.9-31.2) | 24.6(19.7-29.5) |
| Latvia | 23.0(20.6-25.5) | 20.7(17.8-23.5) | 36.8(33.1-40.6) | 19.5(16.6-22.3) |
| Lithuania | 25.1(22.1-28.1) | 19.4(16.9-21.8) | 38.0(34.5-41.4) | 17.6(14.2-21.0) |
| Macedonia | 19.3(16.5-22.2) | 9.7(7.8-11.6) | 32.3(28.4-36.3) | 38.6(32.2-45.1) |
| Malta | 14.0(8.8-19.1) | 7.6(3.6-11.5) | 40.1(32.8-47.4) | 38.4(31.1-45.6) |
| Montenegro | 30.2(26.0-34.4) | 14.5(11.4-17.5) | 29.1(26.3-31.9) | 26.2(21.5-31.0) |
| Poland | 13.7(11.3-16.1) | 15.9(13.4-18.4) | 41.5(38.7-44.3) | 28.8(25.5-32.2) |
| Portugal | 9.5(7.7-11.4) | 19.3(16.3-22.3) | 53.4(50.4-56.3) | 17.8(15.1-20.5) |
| Republic of Moldova | 31.4(26.9-36.0) | 15.4(13.0-17.9) | 30.0(25.7-34.2) | 23.2(18.9-27.4) |
| Romania | 29.1(25.7-32.5) | 14.8(12.7-16.9) | 36.3(33.2-39.4) | 19.8(17.2-22.4) |
| Russian Federation | 24.9(19.1-30.7) | 19.2(15.4-23.0) | 34.1(30.1-38.2) | 21.7(16.5-26.9) |
| San Marino | 8.6(2.7-14.5) | 7.9(2.3-13.5) | 40.3(27.7-52.8) | 43.2(27.6-58.9) |
| Serbia | 24.0(21.5-26.4) | 10.0(8.3-11.7) | 28.0(25.4-30.6) | 38.0(35.2-40.8) |
| Slovakia | 15.7(13.4-17.9) | 20.9(18.7-23.1) | 44.0(41.2-46.8) | 19.4(16.8-22.1) |
| Slovenia | 15.6(11.7-19.4) | 13.0(8.8-17.3) | 43.2(37.3-49.1) | 28.2(19.1-37.4) |
| Tajikistan | 62.3(46.8-77.8) | 6.3(0.1-12.6) | 17.2(6.1-28.3) | 14.2(3.2-25.1) |
| Turkey | 24.9(23.8-26.0) | 17.3(16.2-18.4) | 31.7(30.2-33.2) | 26.1(24.3-28.0) |
| Turkmenistan | 100.0(100.0-100.0) | 0.0(0.0-0.0) | 0.0(0.0-0.0) | 0.0(0.0-0.0) |
| Ukraine | 31.8(26.7-37.0) | 22.5(17.6-27.3) | 34.8(29.2-40.5) | 10.9(8.6-13.2) |
| **South-East Asia** |  |  |  |  |
| Bangladesh | 24.2(10.3-38.1) | 12.1(3.2-21.0) | 54.5(30.8-78.2) | 9.2(0.0-18.6) |
| Bhutan | 13.6(10.1-17.1) | 17.5(13.8-21.2) | 44.1(39.5-48.7) | 24.8(20.5-29.1) |
| Indonesia | 19.6(17.6-21.6) | 28.2(24.8-31.5) | 37.8(35.2-40.5) | 14.5(12.4-16.5) |
| Maldives | 24.1(17.5-30.6) | 14.8(11.1-18.5) | 40.6(33.4-47.8) | 20.6(15.2-25.9) |
| Myanmar | 17.3(12.6-22.0) | 11.0(8.0-14.1) | 41.0(35.5-46.5) | 30.7(24.9-36.6) |
| Nepal | 37.4(27.7-47.2) | 27.7(20.3-35.1) | 15.5(8.2-22.9) | 19.3(10.1-28.6) |
| Sri Lanka | 27.4(17.4-37.3) | 7.0(2.5-11.5) | 30.8(13.5-48.0) | 34.8(14.2-55.5) |
| Thailand | 14.4(8.5-20.2) | 21.3(16.2-26.5) | 39.7(34.3-45.2) | 24.6(20.2-28.9) |
| Timor-Leste | 15.1(11.9-18.3) | 11.7(9.0-14.5) | 37.0(29.8-44.2) | 36.1(29.3-43.0) |
| **Western Pacific** |  |  |  |  |
| Brunei Darussalam | 26.4(20.1-32.8) | 14.4(8.5-20.3) | 37.8(30.5-45.1) | 21.4(15.2-27.6) |
| Cambodia | 29.1(8.8-49.4) | 6.4(0.0-18.7) | 34.1(12.2-56.0) | 30.3(10.8-49.9) |
| Cook Islands | 43.8(35.3-52.4) | 22.3(15.1-29.5) | 21.5(14.5-28.6) | 12.3(6.7-18.0) |
| Fiji | 12.1(6.6-17.7) | 11.3(5.9-16.8) | 28.5(22.0-35.0) | 48.1(40.7-55.5) |
| Guam | 28.4(23.8-33.0) | 20.7(16.0-25.3) | 33.3(28.3-38.3) | 17.6(12.7-22.4) |
| Kiribati | 14.8(10.3-19.4) | 13.0(9.5-16.5) | 40.8(34.5-47.2) | 31.3(24.4-38.2) |
| Laos | 18.0(13.5-22.5) | 20.1(14.8-25.3) | 31.1(26.1-36.2) | 30.8(25.0-36.6) |
| Macao (China) | 41.0(29.4-52.6) | 16.3(10.8-21.7) | 31.2(19.5-42.9) | 11.5(6.8-16.3) |
| Marshall Islands | 14.9(10.2-19.6) | 12.1(6.9-17.3) | 34.7(28.6-40.9) | 38.3(31.0-45.6) |
| Micronesia | 18.6(15.9-21.2) | 14.8(12.4-17.2) | 43.5(40.3-46.7) | 23.2(19.6-26.7) |
| Mongolia | 19.7(16.5-22.9) | 18.7(15.7-21.6) | 39.9(36.9-42.8) | 21.8(18.1-25.4) |
| New Caledonia | 10.0(6.4-13.6) | 22.0(16.0-28.0) | 43.3(38.2-48.3) | 24.7(16.9-32.5) |
| Niue | 18.9(11.9-25.8) | 15.9(8.2-23.6) | 32.7(20.9-44.6) | 32.5(17.3-47.6) |
| Northern Mariana Islands | 22.6(19.9-25.2) | 21.2(16.2-26.2) | 42.6(37.9-47.2) | 13.7(10.6-16.7) |
| Palau | 25.8(21.2-30.4) | 22.6(17.4-27.9) | 43.0(37.3-48.7) | 8.6(5.9-11.4) |
| Papua New Guinea | 11.7(7.6-15.8) | 10.5(7.3-13.7) | 36.3(30.0-42.6) | 41.5(34.7-48.3) |
| Philippines | 27.6(18.9-36.3) | 15.0(11.9-18.1) | 32.0(27.3-36.7) | 25.3(21.1-29.6) |
| Samoa | 24.3(17.9-30.7) | 15.1(5.1-25.1) | 36.3(25.5-47.1) | 24.3(16.4-32.2) |
| South Korea | 7.9(5.5-10.4) | 7.0(4.8-9.3) | 40.6(35.7-45.5) | 44.4(39.4-49.5) |
| Tokelau | 27.5(18.2-36.7) | 36.2(22.7-49.8) | 25.0(8.4-41.6) | 11.3(2.1-20.6) |
| Tonga | 23.7(16.8-30.6) | 19.3(14.5-24.1) | 39.0(29.1-48.8) | 18.0(13.7-22.3) |
| Tuvalu | 30.7(18.7-42.7) | 12.3(7.1-17.5) | 41.2(31.3-51.0) | 15.8(4.9-26.7) |
| Vanuatu | 6.8(3.6-10.0) | 12.0(3.9-20.0) | 23.3(15.1-31.5) | 58.0(47.6-68.3) |
| Viet Nam | 19.1(14.2-23.9) | 13.2(9.0-17.4) | 29.6(21.5-37.7) | 38.2(26.9-49.4) |

Data are presented as % (95%CI).

^a^ United Nations Relief and Works Agency for Palestine Refugees in the Near East.

**eTable 3. Trends in the average age of cigarette smoking initiation among adolescent smoking experiencers in 148 countries/territories** between 1999 and 2020 by country/territory

| **Country/territory** | **Representativeness** | **Survey years** | **First survey, years** | **Last survey, years** | **Absolute change** | **Absolute change per 5-year** | ***P for trend*** |
| --- | --- | --- | --- | --- | --- | --- | --- |
| **Africa** |  |  |  |  |  |  |  |
| Algeria | National | 2007, 2013 | 10.85 | 10.92 | 0.07 | 0.06 | 0.697 |
| Botswana | National | 2001, 2008 | 11.05 | 10.71 | -0.34 | -0.24 | 0.268 |
| Burkina Faso | Subnational | 2001, 2006, 2009 | 11.28 | 10.89 | -0.39 | -0.24 | 0.219 |
| Comoros | National | 2007, 2015 | 11.37 | 11.11 | -0.26 | -0.16 | 0.415 |
| Congo | National | 2006, 2009, 2019 | 11.39 | 10.10 | -1.29 | -0.50 | 0.001 |
| Ghana | National | 2000, 2006, 2009,2017 | 9.48 | 10.19 | 0.71 | 0.21 | 0.173 |
| Kenya | National | 2001, 2007, 2013 | 10.22 | 10.05 | -0.17 | -0.07 | 0.442 |
| Lesotho | National | 2002, 2008 | 11.43 | 10.47 | -0.96 | -0.80 | 0.004 |
| Madagascar | National | 2008, 2018 | 11.98 | 12.14 | 0.16 | 0.08 | 0.662 |
| Malawi | National | 2005, 2009 | 9.59 | 9.24 | -0.35 | -0.44 | 0.727 |
| Mauritania | National | 2001, 2006, 2009, 2018 | 11.18 | 9.63 | -1.55 | -0.46 | <0.001 |
| Mauritius | National | 2003, 2008, 2016 | 11.52 | 11.94 | 0.42 | 0.16 | 0.091 |
| Namibia | National | 2004, 2008 | 11.37 | 10.39 | -0.98 | -1.23 | 0.003 |
| Niger | National | 2001, 2006, 2009 | 11.29 | 11.51 | 0.22 | 0.14 | 0.324 |
| Senegal | National | 2002, 2007, 2013, 2020 | 11.48 | 10.69 | -0.79 | -0.22 | 0.042 |
| Seychelles | National | 2002, 2007, 2015 | 11.60 | 12.15 | 0.55 | 0.21 | 0.022 |
| South Africa | National | 1999, 2002, 2008, 2011 | 11.67 | 11.91 | 0.24 | 0.10 | 0.111 |
| Swaziland | National | 2001, 2005, 2009 | 11.15 | 10.45 | -0.70 | -0.44 | 0.019 |
| Togo | National | 2002, 2007, 2013, 2019 | 10.64 | 10.89 | 0.25 | 0.07 | 0.948 |
| Uganda | National | 2007, 2011, 2018 | 10.21 | 10.05 | -0.16 | -0.07 | 0.865 |
| United Republic of Tanzania | Subnational | 2003, 2008, 2016 | 9.75 | 9.65 | -0.10 | -0.04 | 0.184 |
| Zambia | Subnational | 2002, 2007, 2011 | 10.12 | 10.93 | 0.81 | 0.45 | 0.149 |
| Zimbabwe | Subnational | 1999, 2003, 2008, 2014 | 11.18 | 11.04 | -0.14 | -0.05 | 0.861 |
| **Americas** |  |  |  |  |  |  |  |
| Antigua and Barbuda | National | 2000, 2004, 2009, 2017 | 10.85 | 10.93 | 0.08 | 0.02 | 0.742 |
| Argentina | National | 2007, 2012, 2018 | 12.32 | 12.32 | 0.00 | 0.00 | 0.885 |
| Bahamas | National | 2000, 2004, 2009, 2013 | 11.11 | 10.71 | -0.40 | -0.15 | 0.280 |
| Barbados | National | 1999, 2002, 2007, 2013 | 11.27 | 11.15 | -0.12 | -0.04 | 0.213 |
| Belize | National | 2002, 2008, 2014 | 11.34 | 11.76 | 0.42 | 0.18 | 0.097 |
| Bolivia | Subnational | 2000, 2003, 2012, 2018 | 12.07 | 11.81 | -0.26 | -0.07 | 0.245 |
| Brazil | Subnational | 2002, 2004, 2005, 2006, 2007, 2009, 2011, 2012 | 11.89 | 11.56 | -0.33 | -0.17 | 0.391 |
| Chile | Subnational | 2000, 2003, 2008, 2016 | 11.54 | 12.19 | 0.65 | 0.20 | <0.001 |
| Colombia | Subnational | 2001, 2007 | 11.62 | 11.85 | 0.23 | 0.19 | 0.124 |
| Costa Rica | National | 1999, 2002, 2008, 2013 | 12.02 | 12.03 | 0.01 | 0.00 | 0.782 |
| Cuba | National | 2010, 2018 | 12.24 | 12.48 | 0.24 | 0.15 | 0.352 |
| Dominica | National | 2000, 2004, 2009 | 11.00 | 11.42 | 0.42 | 0.23 | 0.052 |
| Dominican Republic | National | 2004, 2011, 2016 | 11.36 | 11.74 | 0.38 | 0.16 | 0.257 |
| Ecuador | Subnational | 2001, 2007, 2016 | 11.67 | 11.84 | 0.17 | 0.06 | 0.272 |
| El Salvador | National | 2003, 2009, 2015 | 11.69 | 11.79 | 0.10 | 0.04 | 0.567 |
| Grenada | National | 2000, 2004, 2009, 2016 | 10.56 | 11.12 | 0.56 | 0.18 | 0.086 |
| Guatemala | National | 2008, 2015 | 11.78 | 12.12 | 0.34 | 0.24 | 0.001 |
| Guyana | National | 2000, 2004, 2010, 2015 | 10.43 | 10.99 | 0.56 | 0.19 | 0.197 |
| Haiti | Subnational | 2000, 2005 | 11.63 | 11.50 | -0.13 | -0.13 | 0.750 |
| Honduras | National | 2003, 2016 | 11.49 | 11.65 | 0.16 | 0.06 | 0.382 |
| Jamaica | National | 2000, 2006, 2010, 2017 | 10.37 | 11.46 | 1.09 | 0.32 | 0.012 |
| Mexico | Subnational | 2000, 2003, 2005, 2006, 2008, 2011 | 11.90 | 12.14 | 0.24 | 0.11 | 0.647 |
| Nicaragua | National | 2014, 2019 | 11.98 | 11.53 | -0.45 | -0.45 | 0.001 |
| Panama | National | 2002, 2008, 2012, 2017 | 11.85 | 11.87 | 0.02 | 0.01 | 0.924 |
| Paraguay | National | 2008, 2014, 2019 | 11.84 | 11.86 | 0.02 | 0.01 | 0.611 |
| Peru | National | 2007, 2014, 2019 | 12.22 | 12.33 | 0.11 | 0.05 | 0.588 |
| Saint Kitts and Nevis | National | 2002, 2010 | 10.27 | 10.71 | 0.44 | 0.28 | 0.179 |
| Saint Lucia | National | 2000, 2007, 2011, 2017 | 10.79 | 11.21 | 0.42 | 0.12 | 0.029 |
| Saint Vincent and the Grenadines | National | 2000, 2007, 2011, 2018 | 10.41 | 11.17 | 0.76 | 0.21 | 0.014 |
| Suriname | National | 2000, 2004, 2009, 2016 | 11.43 | 11.42 | -0.01 | 0.00 | 0.617 |
| Trinidad and Tobago | National | 2000, 2007, 2011, 2017 | 11.39 | 11.45 | 0.06 | 0.02 | 0.571 |
| Uruguay | National | 2007, 2014 | 12.20 | 12.44 | 0.24 | 0.17 | 0.081 |
| Venezuela | National | 1999, 2010, 2019 | 11.99 | 12.19 | 0.20 | 0.05 | 0.345 |
| **Eastern Mediterranean** |  |  |  |  |  |  |  |
| Afghanistan | Subnational | 2004, 2010, 2017 | 11.18 | 10.62 | -0.56 | -0.22 | 0.985 |
| Bahrain | National | 2002, 2015 | 11.39 | 10.91 | -0.48 | -0.18 | 0.037 |
| Djibouti | National | 2003, 2009, 2013 | 11.94 | 10.68 | -1.26 | -0.63 | 0.008 |
| Egypt | National | 2001, 2005, 2009, 2014 | 10.24 | 10.94 | 0.70 | 0.27 | 0.120 |
| Gaza Strip | Subnational | 2000, 2005, 2013, 2019 | 10.87 | 11.12 | 0.25 | 0.07 | 0.070 |
| Iran | National | 2003, 2007 | 10.97 | 10.29 | -0.68 | -0.85 | 0.044 |
| Iraq | National | 2014, 2019 | 11.02 | 11.34 | 0.32 | 0.32 | 0.100 |
| Jordan | National | 1999, 2003, 2007, 2009, 2014 | 11.10 | 11.59 | 0.49 | 0.16 | 0.041 |
| Kuwait | National | 2001, 2005, 2009, 2016 | 11.61 | 11.51 | -0.10 | -0.03 | 0.476 |
| Lebanon | National | 2001, 2005, 2011 | 11.40 | 11.08 | -0.32 | -0.16 | 0.080 |
| Libyan Arab Jamahiriya | National | 2003, 2007, 2010 | 11.08 | 11.14 | 0.06 | 0.04 | 0.902 |
| Morocco | National | 2001, 2006, 2010, 2016 | 11.49 | 10.87 | -0.62 | -0.21 | 0.104 |
| Oman | National | 2002, 2010, 2016 | 11.23 | 10.85 | -0.38 | -0.14 | 0.404 |
| Pakistan | National | 2004, 2013 | 11.08 | 10.28 | -0.80 | -0.44 | 0.079 |
| Qatar | National | 2004, 2007, 2013, 2018 | 10.99 | 11.10 | 0.11 | 0.04 | 0.986 |
| Saudi Arabia | National | 2007, 2010 | 11.51 | 11.59 | 0.08 | 0.13 | 0.773 |
| Somalia | Subnational | 2004, 2007 | 8.47 | 10.50 | 2.03 | 3.38 | 0.086 |
| Sudan | National | 2001, 2005, 2009 | 11.11 | 10.28 | -0.83 | -0.52 | 0.152 |
| Syrian Arab Republic | National | 2002, 2007, 2010 | 11.67 | 11.62 | -0.05 | -0.03 | 0.860 |
| Tunisia | National | 2001, 2007, 2010, 2017 | 11.36 | 11.00 | -0.36 | -0.11 | 0.037 |
| United Arab Emirates | National | 2002, 2005, 2013 | 11.14 | 11.09 | -0.05 | -0.02 | 0.758 |
| UNRWA^a^ GAZA | Regional | 2008, 2013 | 10.53 | 10.91 | 0.38 | 0.38 | 0.179 |
| UNRWA^a^ Jordan | Regional | 2008, 2014 | 11.60 | 11.59 | -0.01 | -0.01 | 0.971 |
| UNRWA^a^ Lebanon | Regional | 2008, 2013 | 11.64 | 11.33 | -0.31 | -0.31 | 0.261 |
| UNRWA^a^ west bank | Regional | 2008, 2014 | 11.47 | 11.35 | -0.12 | -0.10 | 0.678 |
| west BANK | Regional | 2000, 2005, 2009, 2016 | 11.17 | 11.31 | 0.14 | 0.04 | 0.796 |
| Yemen | National | 2003, 2008, 2014 | 11.24 | 11.70 | 0.46 | 0.21 | 0.003 |
| **Europe** |  |  |  |  |  |  |  |
| Albania | National | 2004, 2009, 2015 | 11.17 | 10.66 | -0.51 | -0.23 | 0.019 |
| Armenia | National | 2004, 2009 | 10.09 | 10.19 | 0.10 | 0.10 | 0.697 |
| Azerbaijan | National | 2011, 2016 | 10.64 | 10.83 | 0.19 | 0.19 | 0.688 |
| Belarus | National | 2004, 2015 | 10.87 | 11.89 | 1.02 | 0.46 | <0.001 |
| Bosnia and Herzegovina | National | 2008, 2013 | 10.48 | 10.88 | 0.40 | 0.40 | 0.027 |
| Bulgaria | National | 2002, 2008, 2015 | 11.51 | 11.98 | 0.47 | 0.18 | 0.035 |
| Croatia (Hrvatska) | National | 2003, 2007, 2011, 2016 | 10.47 | 11.78 | 1.31 | 0.50 | <0.001 |
| Cyprus | National | 2005, 2011 | 11.60 | 12.49 | 0.89 | 0.74 | 0.006 |
| Czech Republic | National | 2002, 2007, 2011, 2016 | 10.81 | 11.60 | 0.79 | 0.28 | <0.001 |
| Estonia | National | 2003, 2007 | 10.36 | 10.47 | 0.11 | 0.14 | 0.313 |
| Georgia | National | 2003, 2008, 2014, 2017 | 9.78 | 10.80 | 1.02 | 0.36 | 0.048 |
| Greece | National | 2005, 2013 | 11.56 | 11.89 | 0.33 | 0.21 | 0.037 |
| Hungary | National | 2003, 2008 | 11.46 | 11.56 | 0.10 | 0.10 | 0.428 |
| Italy | National | 2010, 2014, 2018 | 12.39 | 12.71 | 0.32 | 0.20 | 0.025 |
| Kazakhstan | National | 2004, 2009, 2014 | 10.68 | 10.28 | -0.40 | -0.20 | 0.290 |
| Kosovo | National | 2004, 2016 | 10.87 | 10.96 | 0.09 | 0.04 | 0.652 |
| Kyrgyzstan | National | 2004, 2008, 2014, 2019 | 10.93 | 10.82 | -0.11 | -0.04 | 0.429 |
| Latvia | National | 2002, 2007, 2011, 2014, 2019 | 10.37 | 11.34 | 0.97 | 0.29 | <0.001 |
| Lithuania | National | 2001, 2005, 2009, 2014, 2018 | 10.47 | 11.25 | 0.78 | 0.23 | <0.001 |
| Macedonia | National | 2003, 2008, 2016 | 11.37 | 12.09 | 0.72 | 0.28 | 0.016 |
| Montenegro | National | 2004, 2008, 2014, 2018 | 9.42 | 11.10 | 1.68 | 0.60 | 0.077 |
| Poland | National | 2003, 2016 | 11.06 | 12.08 | 1.02 | 0.39 | <0.001 |
| Republic of Moldova | National | 2004, 2008, 2013, 2019 | 10.04 | 11.10 | 1.06 | 0.35 | <0.001 |
| Romania | National | 2004, 2009, 2013, 2017 | 10.98 | 11.16 | 0.18 | 0.07 | 0.326 |
| Russian Federation | National | 2004, 2015 | 10.66 | 11.33 | 0.67 | 0.30 | <0.001 |
| San Marino | National | 2010, 2014, 2018 | 12.78 | 12.77 | -0.01 | -0.01 | 0.831 |
| Serbia | National | 2008, 2013, 2017 | 10.34 | 11.80 | 1.46 | 0.81 | <0.001 |
| Slovakia | National | 2003, 2007, 2011, 2016 | 10.68 | 11.73 | 1.05 | 0.40 | <0.001 |
| Slovenia | National | 2003, 2007, 2011, 2017 | 11.04 | 12.06 | 1.02 | 0.36 | <0.001 |
| Tajikistan | National | 2004, 2014, 2019 | 9.61 | 9.35 | -0.26 | -0.09 | 0.695 |
| Turkey | National | 2003, 2009, 2012, 2017 | 10.79 | 11.43 | 0.64 | 0.23 | <0.001 |
| Ukraine | National | 2005, 2011, 2017 | 10.56 | 10.72 | 0.16 | 0.07 | 0.470 |
| **South-East Asia** |  |  |  |  |  |  |  |
| Bangladesh | National | 2007, 2013 | 10.40 | 11.16 | 0.76 | 0.63 | 0.270 |
| Bhutan | National | 2004, 2006, 2009, 2013, 2019 | 11.20 | 11.99 | 0.79 | 0.26 | <0.001 |
| India | National | 2006, 2009 | 10.29 | 9.96 | -0.33 | -0.55 | 0.150 |
| Indonesia | National | 2006, 2009, 2014, 2019 | 10.87 | 11.30 | 0.43 | 0.17 | 0.017 |
| Maldives | National | 2004, 2007, 2011, 2019 | 12.02 | 11.41 | -0.61 | -0.20 | 0.543 |
| Myanmar | National | 2001, 2004, 2007, 2011, 2016 | 12.36 | 12.02 | -0.34 | -0.11 | 0.382 |
| Nepal | National | 2001, 2007, 2011 | 11.15 | 10.47 | -0.68 | -0.34 | 0.442 |
| Sri Lanka | National | 1999, 2003, 2007, 2011, 2015 | 10.41 | 11.75 | 1.34 | 0.42 | 0.001 |
| Thailand | National | 2005, 2009, 2015 | 11.92 | 11.89 | -0.03 | -0.01 | 0.489 |
| Timor-Leste | National | 2006, 2009, 2013, 2019 | 11.92 | 12.23 | 0.31 | 0.12 | 0.106 |
| **Western Pacific** |  |  |  |  |  |  |  |
| Brunei Darussalam | National | 2013, 2019 | 11.43 | 11.29 | -0.14 | -0.12 | 0.628 |
| Cambodia | National | 2003, 2010, 2016 | 11.65 | 11.39 | -0.26 | -0.10 | 0.847 |
| China | Subnational | 1999, 2005 | 10.31 | 10.10 | -0.21 | -0.18 | 0.243 |
| Cook Islands | National | 2003, 2008, 2016 | 10.82 | 10.10 | -0.72 | -0.28 | 0.002 |
| Fiji | National | 1999, 2005, 2009, 2016 | 11.68 | 12.63 | 0.95 | 0.28 | 0.003 |
| Guam | National | 2011, 2014, 2017 | 11.38 | 10.98 | -0.40 | -0.33 | 0.054 |
| Kiribati | National | 2009, 2018 | 11.60 | 12.16 | 0.56 | 0.31 | 0.013 |
| Laos | National | 2011, 2016 | 10.00 | 11.75 | 1.75 | 1.75 | <0.001 |
| Macao-China | Regional | 2001, 2005, 2010, 2015 | 10.65 | 10.16 | -0.49 | -0.18 | 0.199 |
| Malaysia | National | 2003, 2009 | 11.74 | 11.32 | -0.42 | -0.35 | 0.030 |
| Marshall Islands | National | 2009, 2016 | 12.49 | 12.25 | -0.24 | -0.17 | 0.519 |
| Micronesia | National | 2007, 2013, 2019 | 11.24 | 11.74 | 0.50 | 0.21 | 0.002 |
| Mongolia | National | 2003, 2007, 2014, 2019 | 11.76 | 11.54 | -0.22 | -0.07 | 0.002 |
| New Zealand | National | 2007, 2008 | 11.35 | 10.81 | -0.54 | -2.70 | 0.388 |
| Niue | National | 2009, 2019 | 10.32 | 11.88 | 1.56 | 0.78 | 0.079 |
| Palau | National | 2013, 2017 | 11.22 | 10.93 | -0.29 | -0.36 | 0.013 |
| Papua New Guinea | National | 2007, 2016 | 12.73 | 12.51 | -0.22 | -0.12 | 0.199 |
| Philippines | National | 2000, 2004, 2007, 2011, 2015 | 12.36 | 11.30 | -1.06 | -0.35 | 0.001 |
| Samoa | National | 2007, 2017 | 11.33 | 11.40 | 0.07 | 0.04 | 0.887 |
| South Korea | National | 2005, 2008, 2013 | 11.26 | 12.83 | 1.57 | 0.98 | <0.001 |
| Tuvalu | National | 2006, 2018 | 11.57 | 10.82 | -0.75 | -0.31 | 0.158 |
| Vanuatu | National | 2007, 2017 | 12.19 | 13.07 | 0.88 | 0.44 | <0.001 |
| Viet Nam | National | 2007, 2014 | 11.67 | 12.02 | 0.35 | 0.25 | 0.195 |

^a^ United Nations Relief and Works Agency for Palestine Refugees in the Near East.
